# Study on time–frequency features of induced charge signals during the damage and failure process of coal medium

**DOI:** 10.1038/s41598-024-59453-1

**Published:** 2024-04-22

**Authors:** Jinguo Lyu, Shixu Li, Yishan Pan, Zhi Tang, Xuebin Wang, Zhanpeng Xue, Yanli Zhang, Yanfang Qiao

**Affiliations:** 1 Ordos Institute, Liaoning Technical University, Ordos, 017000 China; 2 School of Mechanics and Engineering, Liaoning Technical University, Fuxin, 123000 China; 3 College of Mining, Liaoning Technical University, Fuxin, 123000 China; 4 School of Physics, Liaoning University, Shenyang, 110036 China

**Keywords:** Coal medium, Damage and failure, Induced charge signal, Time–frequency features, Geophysics, Solid Earth sciences

## Abstract

Monitoring and preventing coal-rock dynamic disasters are vital for safe mining. To investigate the time–frequency features of induced charge signals with coal damage and failure of roadways, the generation mechanism of free charge in loaded coal is analyzed and the induced charge monitoring test is conducted. According to the stress-induced charge-time curves, the time-domain features of charge signals at each loading stage are obtained. The wavelet threshold denoising approach and generalized Morse wavelet transform method are applied to denoise the raw signals and study the frequency-domain features. Further, the quantitative relationship between the de-noised induced charge signals and the degree of coal damage is established. The results show that the event number, amplitude and fluctuation degree of available induced charge signals are all at a low level in the compaction and elastic stages of the coal, which are mainly generated by the piezoelectric effect and predominantly represent discreteness. When entering the plastic and failure stages, the available signals are primarily produced by the crack propagation and triboelectric effects, with a significant increase in the event number, amplitude, and fluctuation degree. Then the induced charge signals gradually transit from discrete to continuous. Generally, the dominant frequency of the available induced charge signals during the coal damage process is concentrated at 0 ~ 11 Hz. The available induced charge is positively correlated with the degree of coal damage, which can perform the damage degree of coal mass, providing a new approach to evaluate the stability of roadway surrounding rocks.

## Introduction

As coal mines have gradually mined deeper, the typical characteristics of “high ground stress” and “strong mining disturbance” have been highlighted recently. In China, the phenomenon of coal-rock dynamic disasters in underground roadways occurs frequently. Several rockburst accidents have taken place in Henan, Shandong, Liaoning, Hebei, and other mining areas according to incomplete statistics^1,2^, resulting in casualties and equipment damages, which poses a great threat to the safe mining of coal resources. In 2022, the Chinese National Mine Safety Administration (NMSA) lists “strong monitoring” as the primary technical measurement for preventing and controlling underground dynamic hazards. Therefore, searching for a physical quantity to characterize the dynamic damage process of roadway coal-rock and proposing a targeted monitoring method based on its variation rules are becoming urgent needs to ensure safe production.

The birth and occurrence of coal-rock dynamic hazards are accompanied by the changes in physical information, such as drilling cuttings, force, sound, electricity, magnetism, wave velocity, etc. The drilling cutting method^3,4^ indirectly reflects the stress state of the coal seam based on the discharge amount of drilling powder and dynamic phenomenon when drilling, which is simple and practical, but easily affected by manual and has a limited monitoring range. The borehole stress method^5,6^ can continuously monitor the variation of coal-rock stress at multiple points within the influence range of mining stress, which is intuitive, but the monitoring range is also limited and ineffective. The electromagnetic methods include electromagnetic radiation monitoring^7–9^ and electric potential monitoring^10,11^, both of which are used to evaluate the risk of dynamic disasters through monitoring of the electrical or magnetic signals generated by the coal-rock rupture of the roadway. These simple and convenient methods belong to local monitoring but are easily disturbed by the environment. The wave velocity method^12,13^ is intuitiveness and has a moderate monitoring range, indirectly reflecting the degree of stress concentration in the coal-rock medium, but it is hard to achieve continuous monitoring. The acoustic emission method^14–17^ has a wide scope range and belongs to regional monitoring, which mainly adopts microseismic monitoring technique. This method utilizes the spatial and temporal distribution law of energy released by coal rock fracture to search for potential hazards, and it is one of the most effective geophysical monitoring tools so far. However, the signals monitored by the acoustic emission method are basically high-energy low-frequency signals, which makes it difficult to capture the relatively high-frequency micro-fracture signals. Since the complexity of the underground environment, there is electromagnetic interference, high temperature, high humidity, mechanical vibration, and other unfavorable conditions, resulting in the physical precursor information of coal-rock interfered. It is difficult to capture all the characteristics of the disaster signals, which leads to low accuracy of prediction. As a result, new physical parameters or new methods need to be provided urgently for improving monitoring.

For local monitoring of dynamic disasters in roadways, the induced charge monitoring method is a new geophysical monitoring method, which has certain advantages that can sense transient variations of coal-rock surface charge signals in a non-contact way. These variations can describe the damage degree of roadway surrounding rock and also can evaluate the stability of roadways. It has previously been observed that anomalies in electrical signals are generated during rock deformation and failure^18–20^. It is reflected in the emission of high-speed free charges and generation of electrical signals^21–23^ and has similar properties in the deformation and fracture process of coal mass^24–26^. Nowadays, previous studies on the induced charge signal monitoring test of coal rock have been conducted and many achievements have been acquired. In terms of test methods, the coal rock will produce induced charge signals with different fluctuation levels under friction, tension, shear, and different deformation and failure modes^27–29^. Additionally, loading conditions^30,31^ and loading paths^32^ also affect the duration and distribution characteristics of induced charge signals. In the aspects of test materials, the decrease of water content, the increase of coal-rock ratio, and the increase of outburst proneness will lead to the increment of the signal richness^33–35^. Besides, the decrease in the crack angle will make the high-value signal appear in advance^36^ and the increase in metamorphic grade will cause the surface charge of coal to show a “V” type change^37^. Moreover, the increase in gas pressure will reduce the peak value of induced charge signals^38^, and the lithology change in coal rock will significantly affect the response regularity of signals^39^, etc. In addition to laboratory research, the induced charge monitoring equipment for complex environments is developed constantly^40–43^, and prediction of rockburst risk in stope based on induced charge monitoring technology is also provided^44,45^.

Although the induced charge monitoring method has developed in the application of roadway surrounding rock stability, the relationship between the damage of coal medium and the time–frequency features of induced charge signals is still unclear to establish, which results in the poor effect of evaluating the degree of coal damage. Therefore, a study on the evolution laws and main features of induced charge signals in time and frequency domains is carried out during the damage and failure process of coal. In previous studies, the Fourier transform is usually used to analyze the frequency domain patterns of induced charge signals, which is generally suitable for processing stable signals but difficult to explore the time–frequency details of non-stationary signals^46^. At the same time, the time–frequency localization ability of wavelet transform has aroused extensive attention from scholars, and it has achieved some results in the analysis of acoustic emission and microseismic signals in coal-rock failures^47–50^. In particular, the wavelet transform is also highly adaptable to analyze and process the non-stationary induced charge signals. With the development of wavelet analysis methods in recent years, analytical wavelets with complex values are proposed, such as Generalized Morse Wavelet (GMW). When using GMW to perform continuous transformation, it can effectively separate the phase and amplitude inside the signals^51,52^, providing support for the accuracy of wavelet analysis.

In summary, based on the generation mechanism of free charge and the principle of induced charge signal monitoring, a uniaxial compression test of induced charge signals monitoring during the coal damage and failure is conducted. The method for recognizing continuous and discrete types of induced charge signals is proposed. Then wavelet threshold denoising method is used to filter and de-noise the signals. Followed by the method of continuous GMW transform, the evolution pattern and primary features in the time–frequency domain of induced charge signals during the damage and failure process are clarified. Subsequently, the quantitative relationship between coal damage and induced charge signals is established to propose the damage factor based on charge accumulation, which provides a new approach to evaluate the damage degree of coal mine roadways.

## Free charge generation mechanism of loaded coals

**Figure 1:**
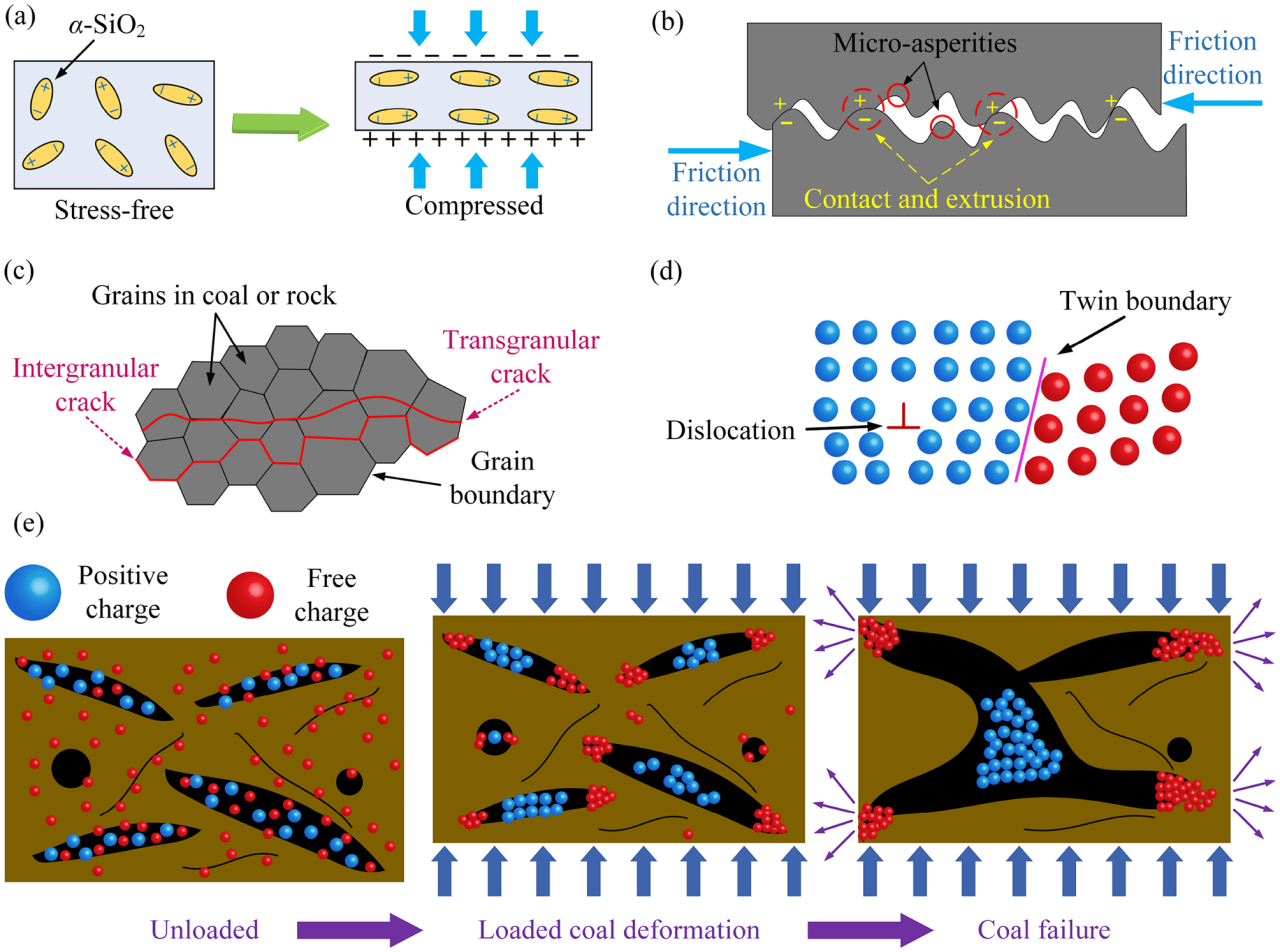
Free charge generation mechanism. (**a**). Piezoelectric effect. (**b**). Triboelectric effect. (**c**). Crack propagation effect. (**d**). Crystal defect effect. (**e**) crack tip discharge effect.

According to previous research studies, the generation mechanism of free charges mainly includes four aspects, which are the piezoelectric effect, triboelectric effect, crack propagation effect, crystal defect effect, and crack tip discharge effect (Fig. 1).

1. Piezoelectric effect. Some solid materials contain piezoelectric bodies, which can cause positive and negative centers to be misaligned due to deformation when subjected to mechanical forces, resulting in a crystal moment that is no longer zero, causing charges to appear on the corresponding surface. Through analysis of coal ash, it is determined that *α*-SiO_2_ minerals naturally exist in coal. As a typical piezoelectric body, *α*-SiO_2_ can generate charges when the coal is loaded^53–56^.
2. Triboelectric effect. Different strengths of micro-asperities are distributed unevenly on the surface and interior of coal. Under stress loading, the contact surface rapidly separates, and the micro-asperities on the contact surface undergo contact and extrusion, causing charge separation and transfer, resulting in opposite electrical properties on the friction surfaces on both sides^57–60^.
3. Crack propagation effect. Coal is a heterogeneous material composed of macromolecules, mineral particles, and cementation. It has distinctive physicochemical properties and consists of some micro-defects such as cracks and joints, leading to non-uniform deformation under stress imposed, resulting in crack propagation and convergence. From a microscopic perspective, intergranular and transgranular fractures can lead to charge separation with the crack developing. The intergranular fracture refers to the gradual fracture alongside the facet of the particle prompted by nearby stress heterogeneity, which destroys the intermolecular force and leads to electric potential changes. However, the transgranular fracture refers to the fracture that not solely proceeds alongside the edge but penetrates the complete particle to smash the internal structure, resulting in free charges^61–64^.
4. Crystal defect effect. Polycrystalline materials within coal often exhibit various crystal defects, which can lead to charge separation upon stress loading. Dislocations, as a kind of linear defects, can cause atomic rows to slip relative to each other and generate cation vacancies, resulting in charge separation. Grain boundaries can also considered a type of planar defect, where stress concentration is first experienced at the grain boundary, causing chemical bonds to destroy and resulting in charge separation^65,66^.
5. crack tip discharge effect. In the unloaded state, the coal system remains in thermal equilibrium, with free electrons undergoing random motion without any directional movement. However, as stress levels rise, the coal experiences loading and deformation. The presence of the tip effect leads to the continuous extension of numerous free charges towards the crack tip, necessitating supplementation from distant free charges. Consequently, the amplitude of the induced charge signal undergoes a substantial increase. Upon the convergence and propagation of internal cracks, resulting in macroscopic damage to the coal, the free charges accumulated at the crack tip due to diffusion are forcefully ejected^67^.

**Figure 2:**
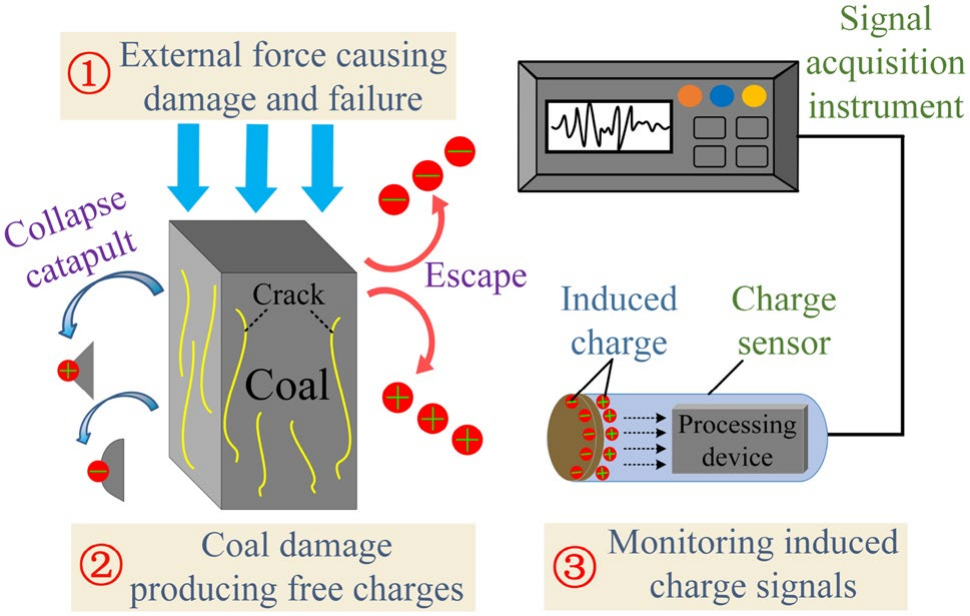
Monitoring process of induced charge signals.

The mechanism of free charge generation shows that internal micro-defects continue to enhance and accumulate when the coal is loaded by exterior pressure, and the degree of damage will increase continuously as the loading progresses. The damage process is frequently accompanied by free charge and collapse of charged coal particles^41^. During the damage and failure process of the coal, the charge sensor can dynamically sense the free charge then output the induced charge signal through the built-in signal processing device and be received by the signal acquisition instrument. The monitoring process of induced charge signals is shown in Fig. 2.

## Monitoring test of induced charge signals during the damage and failure process of loaded coal

**Figure 3:**
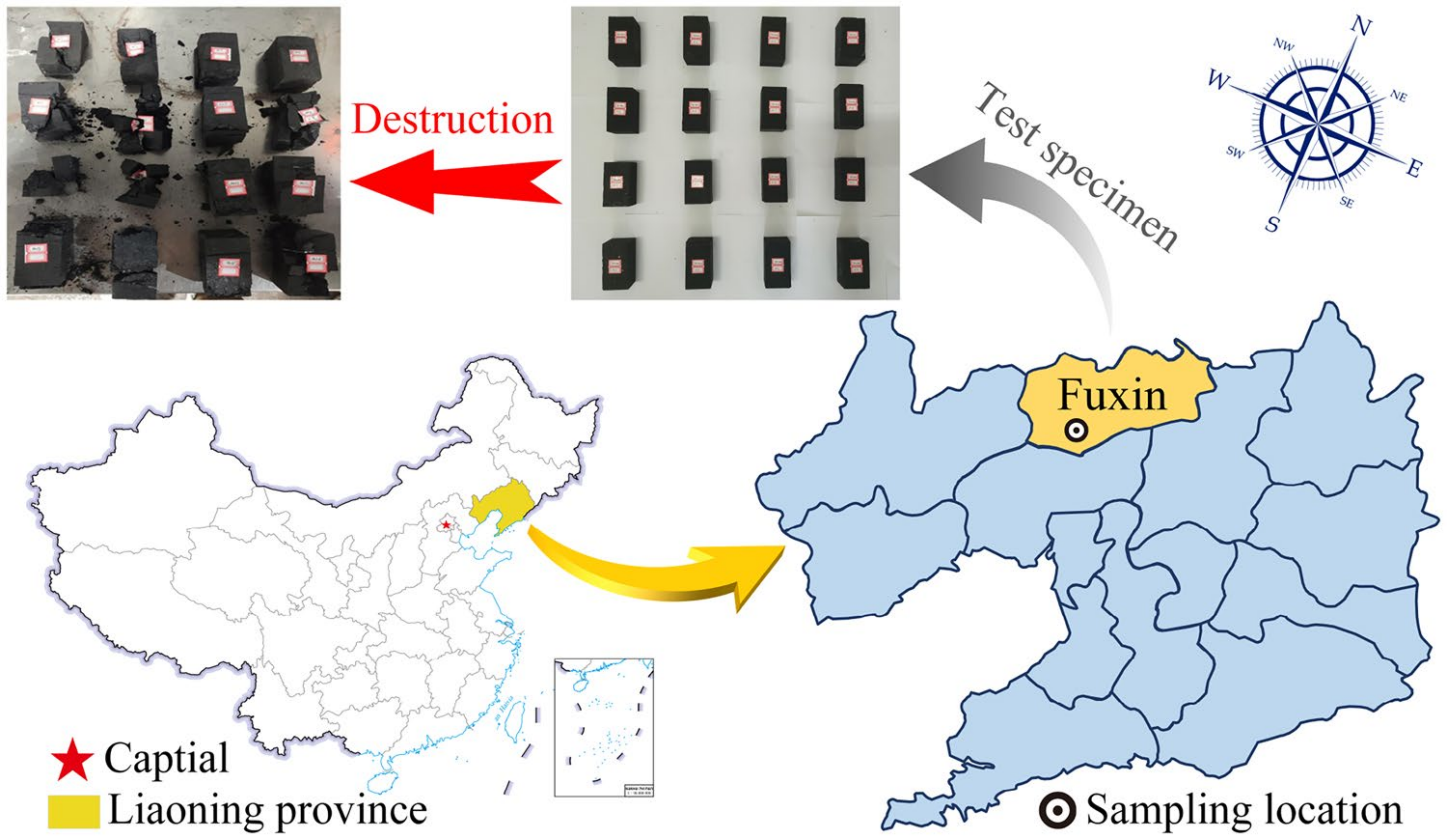
Sampling location of coal samples.

To study the time–frequency features of induced charge, the induced charge signal monitoring test in the condition of uniaxial compression is conducted on coal samples. The coal samples are retrieved from a coal mine in Fuxin City, Liaoning Province (Fig. 3). To ensure the validity of the statistical data and minimize test errors, coal samples were chosen from the same coal block, possessing a smooth surface and no visible cracks. Additionally, the bedding direction was oriented perpendicular to the loading direction. The massive samples are prepared into cuboid samples with the standard size of 50 mm × 50 mm × 100 mm (length × width × height), and its physical parameters are shown in Table 1.

**Table 1:** Physical parameters of coal specimen.

Density/(kg m^−3^)	Compressive strength/MPa	Elastic modulus/GPa
1176.28	6.36	0.66

**Figure 4:**
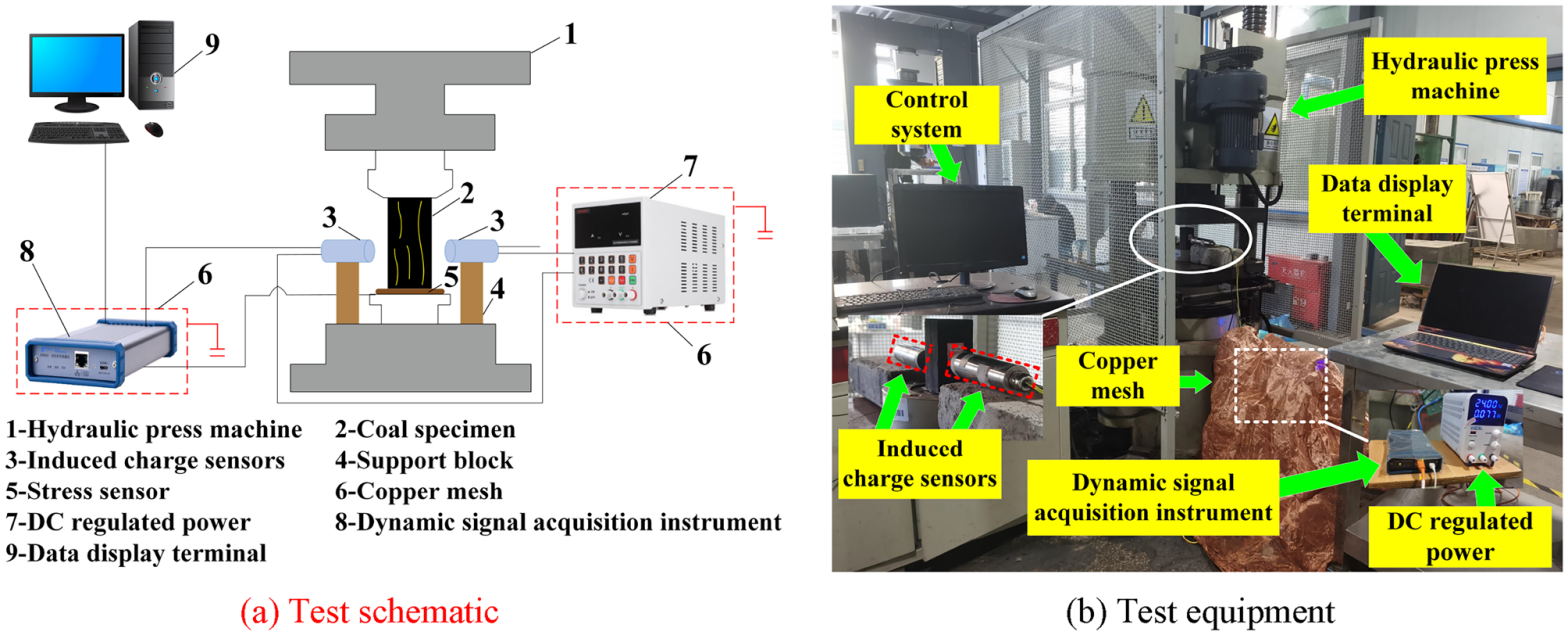
The monitoring test of induced charge in coal uniaxial compression process.

The test is composed of the stress loading system, the data acquisition system, and the system of shielding external signals, as shown in Fig. 4. The stress loading system is a YAW-2000 hydraulic press machine with a maximum axial compression load of 2000 kN. During the test, the displacement loading mode is adopted with a displacement loading velocity of 0.005 mm/s. The data acquisition system mainly includes the stress sensor, the non-contact induced charge sensors, the dynamic signal acquisition instrument, and the data display terminal. The maximum range and minimum resolution of the induced charge sensors are ± 16.5 pC and 0.01 pC, respectively. Moreover, the stress sensor can achieve a maximum stress of 200 kN, meeting the requirements of this test. Through the dynamic signal acquisition instrument, the induced charge sensors and stress sensors are connected to the signal analysis system to monitor and store the charge and stress. The induced charge sensors are arranged 25 mm away from both sides of the specimen and the sampling frequency is set to 1 kHz. The system of shielding external signals utilizes copper mesh to shield the DC regulated power supply and signal acquisition instrument to minimize external noise interference. After conducting the pre-tests, it was found that the electromagnetic shielding effect reached a maximum of 50 dB, which achieved the conditions of this test. The laboratory temperature is 26 °C.

## Results

### Time domain features of induced charge signal during the damage and failure process of coal medium

**Figure 5:**
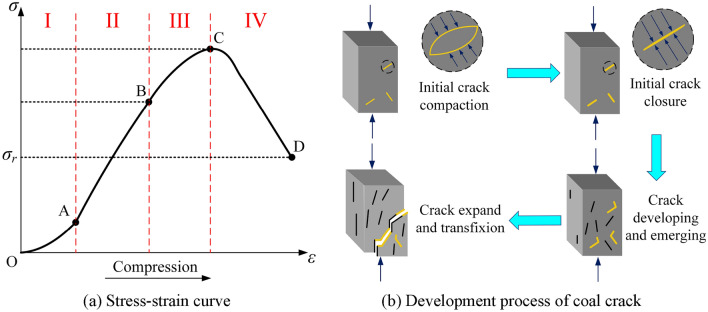
Typical stress–strain curve of the coal and its crack evolution process.

Essentially, the damage and failure process of coal is the evolution of a large number of micro-defects within the coal under stress loading, and the stress–strain curve can well describe this process. Figure 5a is the typical stress–strain curve during the uniaxial compression process of the coal. According to the variation of the curve, the compression process is divided into four stages with different characteristics. I ~ IV are the compaction stage (OA section), elastic stage (AB section), plastic stage (BC section), and failure stage (CD section), respectively. The *σ*_*c*_, *σ*_*e*_, and *σ*_r_ in the Fig. 5a represent peak strength, yield strength, and residual strength of the coal. The evolution of crack development at each stage is shown in Fig. 5b, and the time and frequency domain features of induced charge signal can be different by coal damage and failure evolving.

**Figure 6:**
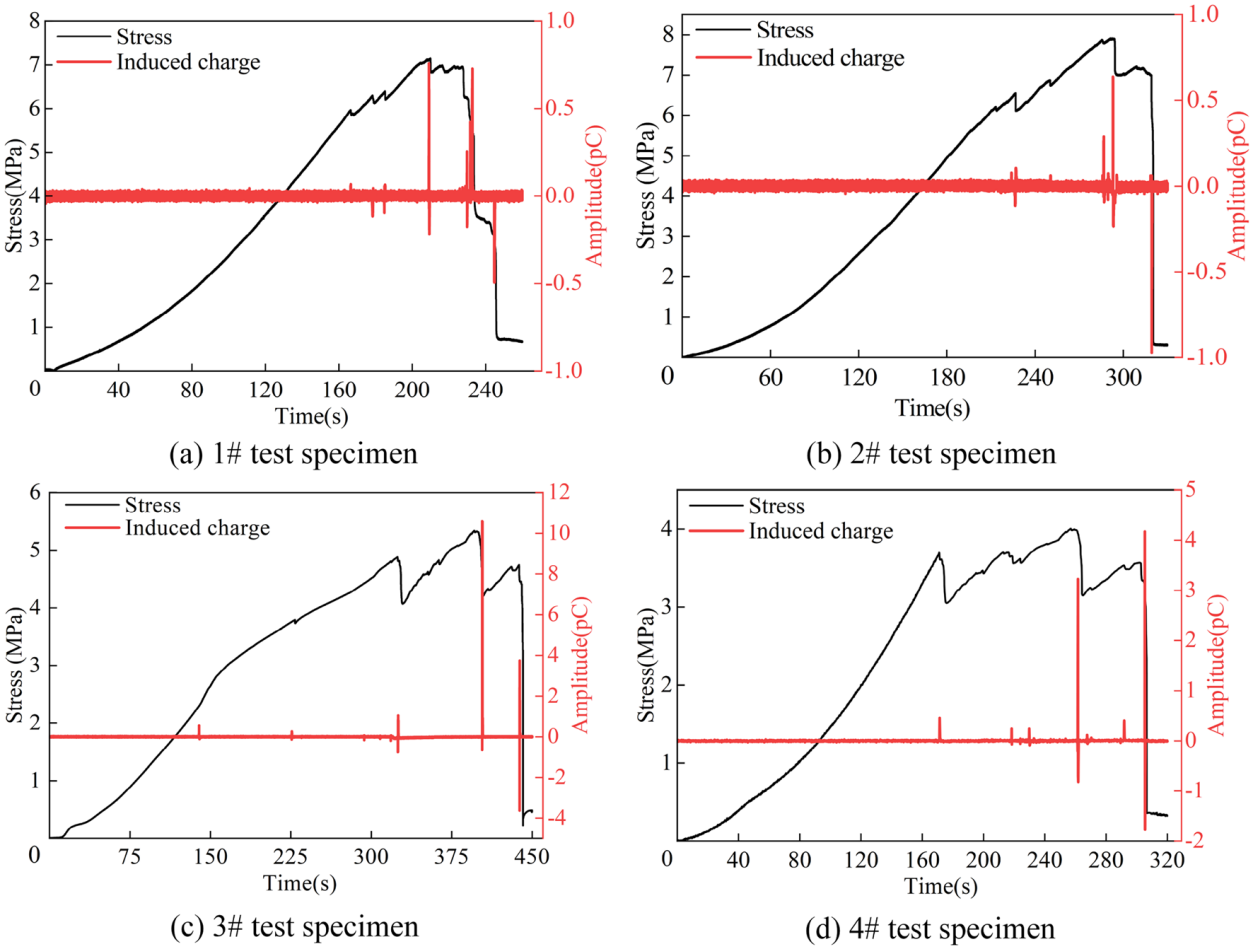
Stress-induced charge-time curve of the coal specimen under uniaxial compression.

The representative stress–strain curve and time series curve of the induced charge signal are selected (Fig. 6). Although the copper mesh can shield interference signals and enhance the identification of induced charges throughout the whole test, some non-charge noise signals are still unavoidably collected. To reveal the time–frequency evolution law of induced charge signal and to clarify the characteristic variations during the coal instability process, the available induced charge signals should be identified in the time domain.

**Figure 7:**
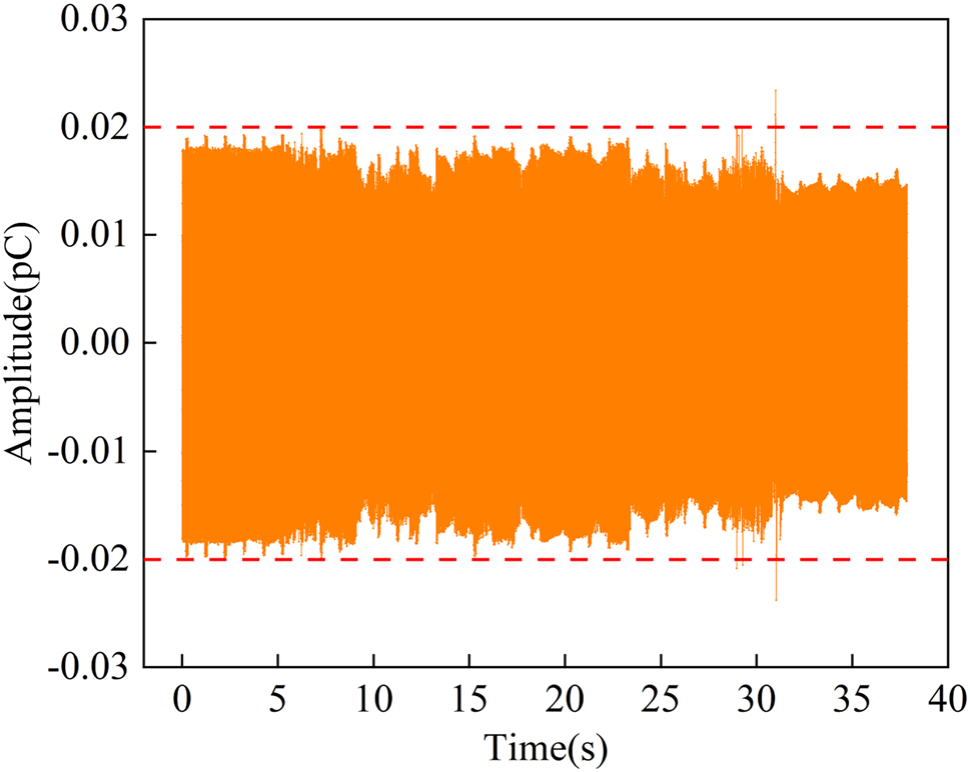
Time-domain waveform of noise signal in laboratory.

By monitoring the unloaded signal in the shielding environment of the ground laboratory, it is observed that the signal fluctuation range is almost concentrated between − 0.02 and 0.02 pC, indicating the signal in this amplitude range could be considered noise interference (Fig. 7). Therefore, the amplitude threshold can be set at 0.02 pC, meaning the raw induced charge signals with an amplitude above 0.02 pC are available charge signals.

In addition, a time series dispersion measurement method based on interquartile range (*IQR*) is used to identify the type of induced charge signals: dividing the induced charge signals according to the unit time (1 s) and acquiring the time series of available charge events; sorting and segmenting the sequence into four equal parts by the ascending order of events. The values at three segment points are the quartiles and marked as *Q*_1_, *Q*_2_, *Q*_3_ from the order of small to large, accordingly *IQR* is the range that obtain the 25% ~ 75% of the data as Eq. (1)^68^.

1 $$IQR = Q_{3} - Q_{1}$$

**Figure 8:**
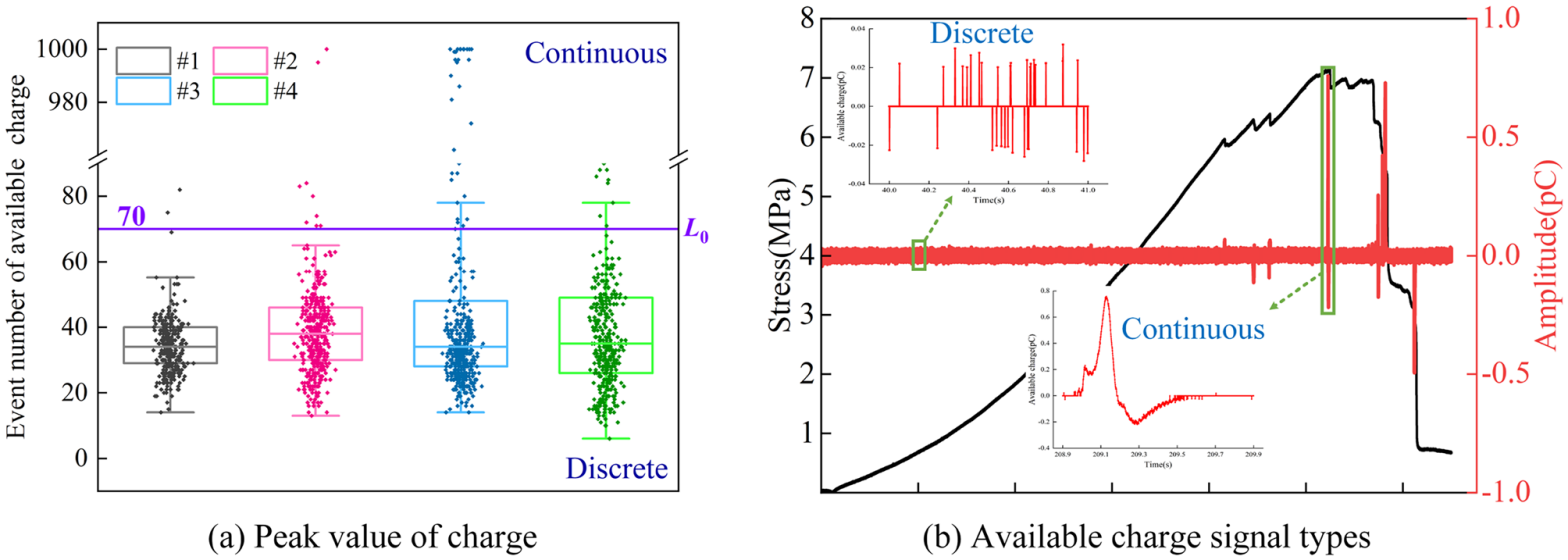
Time-domain definition of available charge.

The *IQR* can identify the dispersion points in the sequence of available charge event numbers, thereby calculating the number of available charge events of each specimen during the whole loading process, as shown in Fig. 8a. Every single point in the boxplot is the number of available charge events per second.

Therefore, the upper and lower limits of the available charge event sequence can be expressed by Eq. (2), within the limits is the main distribution range of the data, and the data outside the limits can be considered as outliers with different properties from the majority^68^.

2 $$\begin{gathered} L_{\max } = Q_{3} + 1.5IQR \hfill \\ L_{\min } = Q_{1} - 1.5IQR \hfill \\ \end{gathered}$$

where *L*_max_ indicates the upper limit and *L*_min_ represents the lower limit.

The average upper limit *L*_0_ of the available charge event number can be calculated as Eq. (3) according to the upper limit of the event number distribution of *N* specimens.

3 $$L_{0} = \frac{1}{N}\sum\limits_{1}^{N} {L_{\max } } \, (N = 1,2,3, \ldots )$$

Calculated by the above equation to get the value of *L*_0_ is 70, and the type of induced charge signal can be classified accordingly. That is the signals whose available charge events are equal to or less than 70 times per second are discrete, and the signals whose events are greater than 70 times per second are continuous instead. Discrete and continuous signals are shown in Fig. 8b.

**Figure 9:**
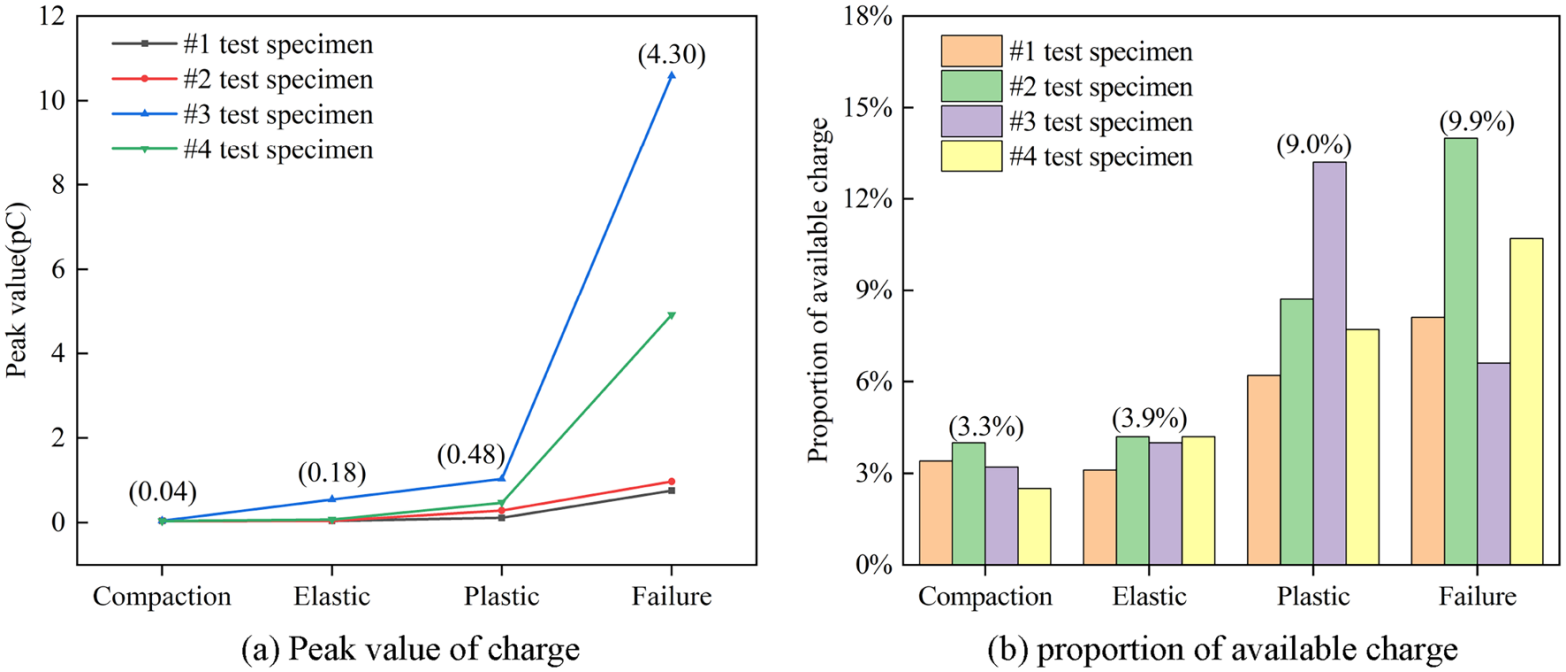
Time-domain parameters statistics of induced charge signal during damage and failure process of coal specimens. (The contents in brackets are the average values at each loading stage).

By analyzing the stress-induced charge-time curve, counting the event number of available induced charge signal and their amplitude changes during the damage and failure process of the coal specimens (Fig. 9), it can be found the following time-domain features of the available induced charge signals at each loading stage:

1. Within the compaction stage at the early loading stage, the initial micro-cracks are gradually compressed and closed. At this time, merely a small amount of available charge signals can be monitored, with the highest induced charge amplitude averaging 0.04 pC and the average number of available charge event ratios accounting for 3.3%. The amplitude and events of available charge signals are at a low level, mainly presenting as discrete induced charge signals.
2. During the elastic stage, the stress–strain curve of the coal approximately exhibits the linear relationship. The average amplitude of the highest induced charge signal is 0.18 pC, and the average proportion of available induced charge events is 3.9%. The amplitude and event number remain at a low level, but they have increased slightly compared with the former stage. Stress drop may also occasionally be generated, indicating the cracks inside the coal occur, accompanied by high amplitude signals. The induced charge signals are still predominantly discrete.
3. Entering the plastic stage, the damage and failure degree of the coal increases with the growth of stress. Meanwhile, the primary and newly-formed cracks consistently expand and converge to the sliding surface until reaching the peak strength of the coal. Compared with compaction and elastic stages, the frequency and magnitude of stress drop increases significantly, and the volatility and amplitude of the available charge signals rise sharply. The average highest amplitude and event proportion of available signals are 0.48 pC and 9.0%, respectively. The signal type gradually transitions from discrete to continuous.
4. In the failure stage, the cracks penetrate to form the macroscopic fracture surface and the main destruction happens rapidly. This stage has the maximum frequency and magnitude of stress drop within the entire coal damage process, meaning the damage and failure degree are the most severe. The available induced charge signals can be monitored abundantly, which have the maximum amplitude and event numbers of induced charge signals during this time. The average maximum amplitude is 4.3 pC, and the average event proportion is 9.9%. Correspondingly, the signals are mainly continuous.

In summary, the process of coal damage and failure can be regarded as the evolution of crack development, emergence and propagation, accompanied by triboelectric and other electrification effects, which will generate free charge of the coal. Increased levels of damage normally produce higher amounts of charges, resulting in the high amplitude induced charge signals that can be monitored.

### Frequency domain features of induced charge signal during the damage and failure process of coal medium

#### De-noising process of induced charge signal based on wavelet thresholding method

Wavelet transform uses mother wavelet functions to decompose signals across time and frequency. If the function *ψ*(*t*) is a square-integrable function *ψ*(*t*) ∈ *L*^2^(*R*). Furthermore, this function must satisfy the admissibility condition (*C*_*ψ*_) and can be used as a mother wavelet function, as presented in Eq. (4)^46^.

4 $$C_{\psi } = \int\limits_{R} {\frac{{\left| {\psi \left( \omega \right)} \right|^{2} }}{\left| \omega \right|}} {\text{ d}}\omega < \infty$$

where *ψ*(*ω*) is the Fourier transform of the mother wavelet *ψ*(*t*).

Then the process of wavelet transform is shown in Eq. (5)^52^.

5 $$\begin{aligned} Wf\left( {u,s} \right) & = \left\langle {f\left( t \right),\psi_{u,s} \left( t \right)} \right\rangle \\ \, & = \frac{1}{\sqrt s }\int\limits_{R} {f\left( t \right)\overline{\psi }\left( {\frac{t - u}{s}} \right)} {\text{ d}}t \\ \end{aligned}$$

where *u* and *s* are used to represent the translation and scale, respectively. The translation parameter allows controlling the time domain localization, while the scale parameter controls the frequency localization. $\overline{\psi }$ is the conjugate of function *ψ*. The commonly used wavelet transform methods include continuous wavelet transform (CWT), discrete wavelet transform (DWT), etc., which essentially selects different translation and scale parameters continuously or discretely to obtain higher time–frequency resolution.

**Figure 10:**
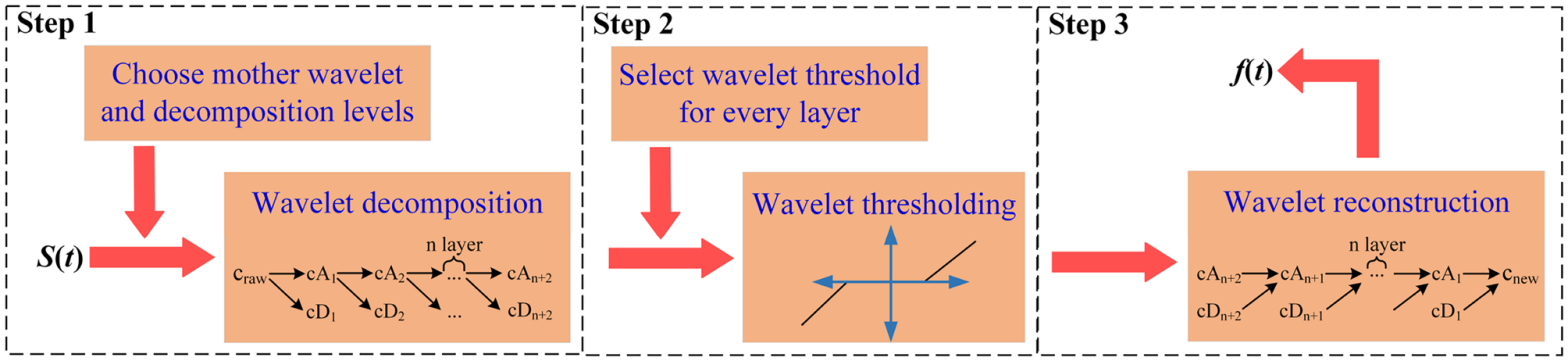
Wavelet de-noising process.

The wavelet threshold denoising is used to remove the noise for analyzing the frequency-domain features of the induced charge signals, and the de-noising process is as follows (Fig. 10):

**Step 1**: Decompose the raw signal. Choose a suitable mother wavelet function to perform *N*-layer DWT for the original induced charge signal *s*(*t*), which acquires the low-frequency coefficients (cA) and high-frequency coefficients (cD) of each layer. The mother wavelet functions are commonly used in the wavelet thresholding method, including Daubechies wavelet, Coiflet wavelet, Symlet wavelet, and Biorthogonal wavelet, etc. Generally, the orthogonality, compactness, symmetry, and regularity of the wavelet properties are required when selecting the mother wavelets. In this paper, the bior5.5 wavelet is selected which is symmetric, compactly supported, and biorthogonal. For the wavelet decomposition layers, the larger the number of decomposition layers, the more obvious the different characteristics of the signal and noise, resulting the signal distortion. Normally, the number of decomposition layers is selected as 3 to 5 in practical applications^69,70^, and a 5-layer decomposition is applied in this study.

**Step 2**: Thresholding the wavelet coefficients for each decomposed layer. Common thresholds include Rigrsure threshold, Heursure threshold, Sqtwolog threshold, Minimaxi threshold, Universal threshold, etc. Once choosing the appropriate threshold, set the threshold for the wavelet coefficients in each layer. The high wavelet coefficients are set to zero but the low coefficients can be preserved after processing. There are two commonly used thresholding methods: hard thresholding and soft thresholding, which both have advantages and disadvantages. Hard thresholding method can preserve the peak characteristics of the signal but introduces discontinuities, while soft thresholding method can smooth the signal but may introduce distortion^71^. To evaluate the preferable selection of threshold and its thresholding method, the Signal to Noise Ratio (*SNR*) of de-noised induced charge signals has been calculated in Eq. (6).

6 $$SNR = 10\log_{10} \frac{{P_{s} }}{{P_{n} }}$$

where *P*_*s*_ is the average power of the raw signal; *P*_*n*_ is the average power of the noise, which is calculated by subtracting the de-noised signal from the raw signal.

**Step 3**: Wavelet reconstruction. The de-noised signal *f*(*t*) is obtained by using the high-frequency and low-frequency coefficients of each decomposition layer.

**Figure 11:**
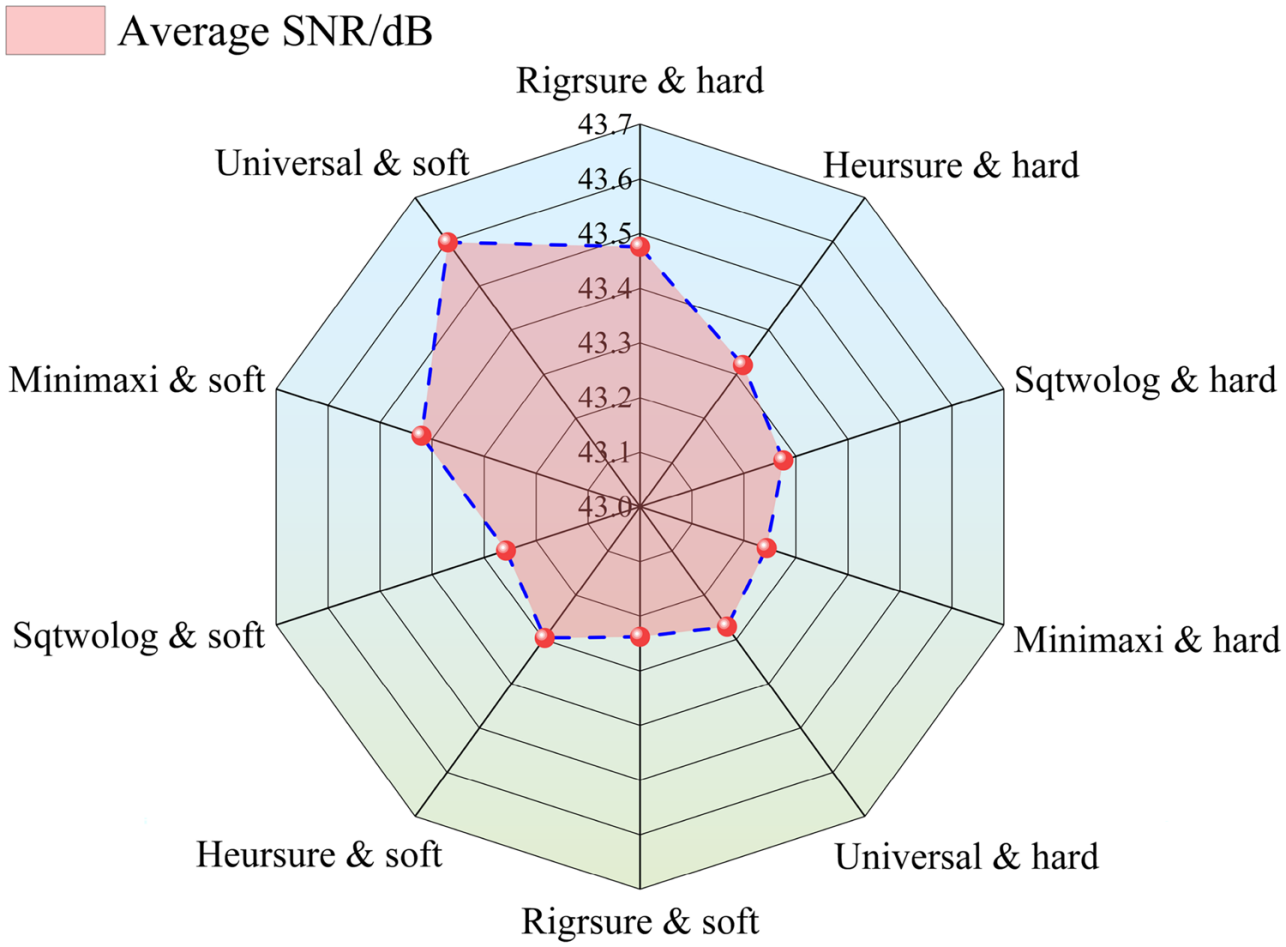
The SNR of induced charge signal of each specimen after processing by amplitude normalization and different wavelet thresholding methods.

The average *SNR* of induced charge signals is calculated after amplitude normalization and applying multiple thresholding methods (Fig. 11). Selecting the Universal threshold and using the soft thresholding method can acquire the better de-noising effect of induced charge signals, which can reach the highest *SNR* of 43.6 dB. The Universal threshold *δ* is proportional to the estimated standard deviation *σ* of the signal noise, and *σ* can be estimated from the set of wavelet coefficients at the highest scale level, as depicted in Eq. (7).

7 $$\delta = \sigma \sqrt {2\log \left( {{\text{length}}\;\left( {{\text{signal}}} \right)} \right)}$$

**Figure12:**
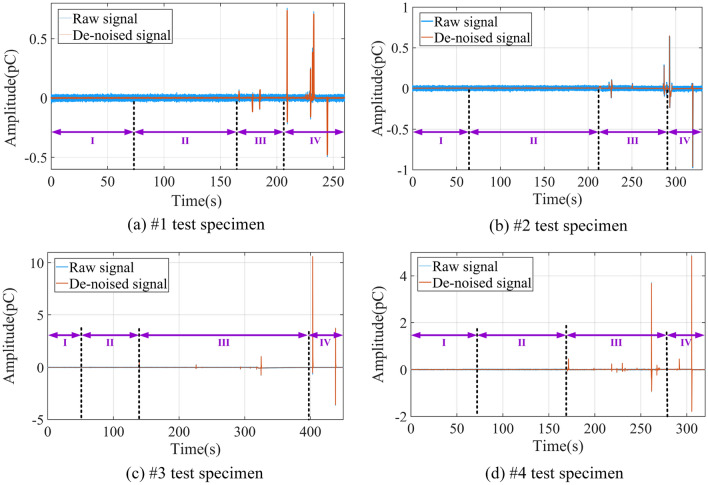
Raw and de-noised induced charge signals (The Roman numerals in the figure refer to the prementioned loading stage).

The raw and de-noised induced charge signals are shown in Fig. 12.

#### Frequency domain features of induced charge signal based on continuous GMW transform

With further study of wavelets, other relevant papers have proposed analytical wavelets including GMW and complex Morlet wavelet^52^. Analytical wavelets have no negative components in frequency support interval, avoiding the artifacts and interferences that may exist in the CWT, which reflects the analyticity^72^. Since GMW has better low-frequency resolution compared with the complex Morlet wavelet, it is more suitable for studying the induced charge signals. Therefore, the continuous GMW transform is used to analyze the frequency-domain features of signals in this study.

The GMW is expressed by Eq. (8) in frequency domain^51^.

8 $$\psi_{{_{\beta ,\gamma } }} \left( \omega \right) = \int\limits_{R} {\psi_{\beta ,\gamma } \left( t \right)} \, e^{ - iwt} dt = 2H\left( \omega \right)\left( {\frac{e\gamma }{\beta }} \right)^{\beta /\gamma } \omega^{\beta } e^{{ - \omega^{\gamma } }}$$

where *ψ*_*β,γ*_(*t*) is time-domain function of GMW, *β* and γ are the parameter that control wavelet attenuation in the time and frequency domain, respectively, which must be greater than zero to make the GMW valid. Moreover, *e* is the Euler number, and the *H*(*ω*) is the Heaviside step function.

The Heaviside step function* H*(*ω*) is defined as Eq. ^9^.

9 $$H\left( \omega \right) = \left\{ {\begin{array}{*{20}c} {0,} & {\omega < 0} \\ {1/2,} & {\omega = 0} \\ {1,} & {\omega > 0} \\ \end{array} } \right.$$

According to the Eq. (8), GMWs are mainly controlled by parameters *β* and γ*.* Multiple analytic wavelets with different characteristics can be obtained by adjusting these two essential parameters. In this work, due to the widespread use of parameters *β* and *γ* as 20 and 3 in fluid dynamics, vibration analysis and system analysis, we choose this typical parameter here^73^. Thus, the obtained GMWs are both Gaussian and symmetric, with good time–frequency resolution ability.

**Figure 13:**
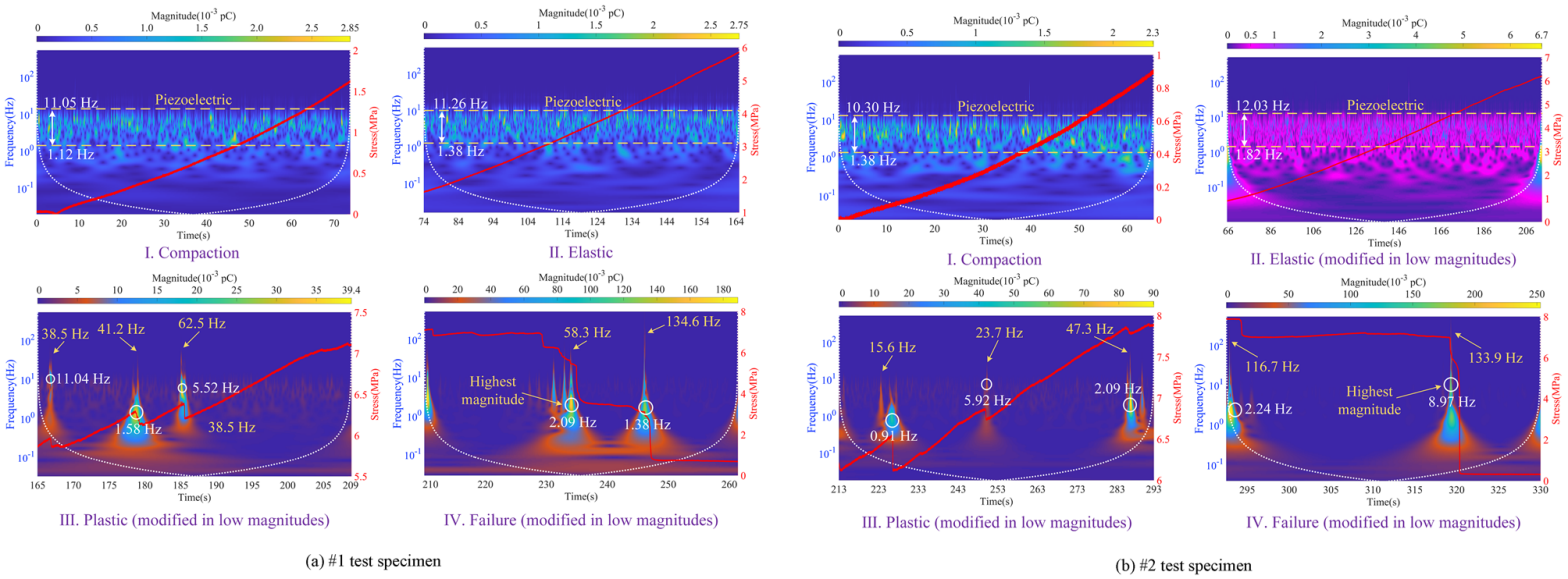

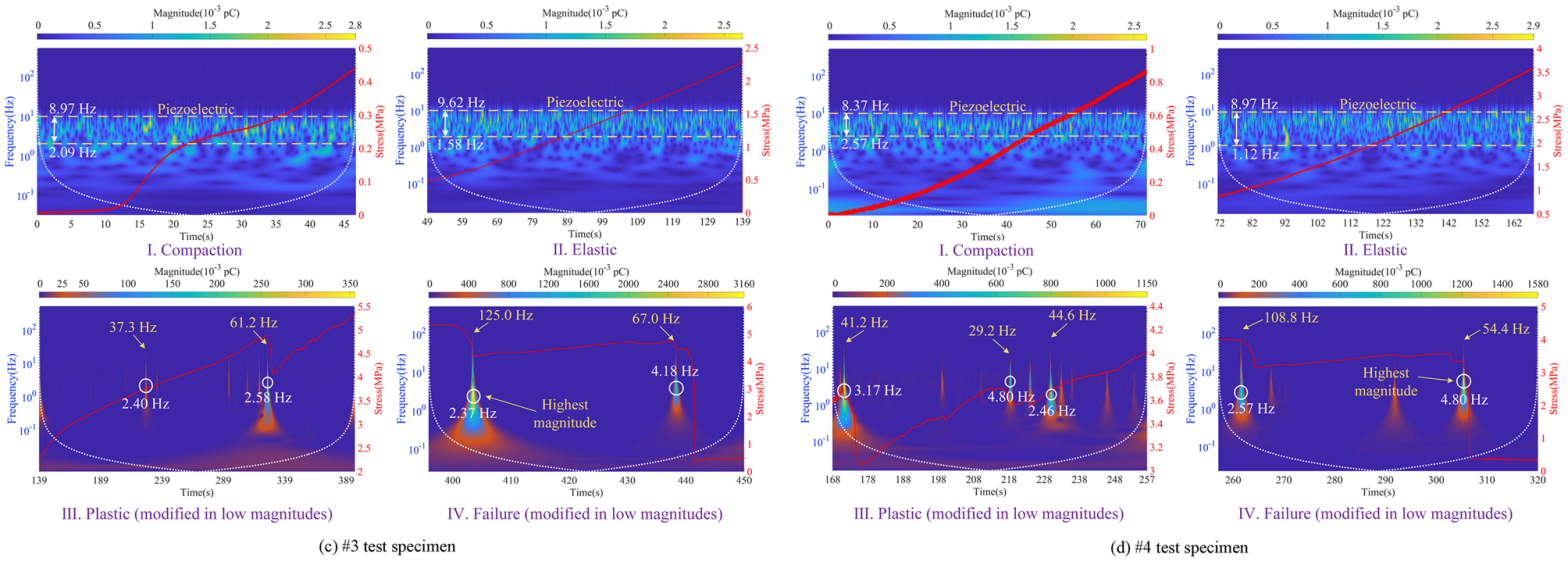
Time–frequency spectrogram of induced charge signals and corresponding stress curve.

Figure 13 is the time–frequency spectrogram of the de-noised induced charge signal, which shows the corresponding time-stress curve of the coal in four stages simultaneously. The region within the white dashed line in the figure indicates the undistorted part of the signal, which can be considered the most accurate representation of the time–frequency information. The yellow arrow represents the highest frequency of the induced charge signal, and the white circle shows where the dominant frequency of the signal is. And the magnitude of the induced charge signal refers to the sum of its absolute amplitude. Therefore, the frequency-domain features of the induced charge signal are as follows:

1. Within the compaction stage, the frequency band of available induced charge signals is mainly concentrated in 1 ~ 11 Hz, with signal magnitudes of 0.001 ~ 0.003 pC, which produces a small amount of charge. The available charge signals within this stage are mainly generated by the piezoelectric effect, suggesting that piezoelectric is not responsible for the generation of the high-valued induced charge signals.
2. During the elastic stage, the frequency-domain features are essentially similar to those of the compaction stage, but the signal distribution is denser. Due to the relatively high amplitude induced charge signals shown at the beginning and end of this stage, low amplitude signals generated by the piezoelectric effect can be clearly observed after chromatographic processing in the #2 specimen.
3. Entering the plastic stage, the dominant frequency of available induced charge signals is 0–11 Hz, but high-frequency signals can also occur, with a maximum frequency of 62.5 Hz. The amplitude of signals is 0.01 ~ 0.5 pC. However, a higher amplitude signal of 1.15 pC is observed in specimen #4 because of the coal variability. These low-frequency induced charge signals have a longer duration and higher amplitude, while high-frequency signals have a shorter duration and lower amplitude.
4. In the failure stage, the dominant frequency of available charge signals is 0–9 Hz, and the duration of low-frequency signals is longer than that of the plastic stage. In addition to the dominant frequency, the frequency limit of available charge signals is higher, which is up to 135 Hz. The amount of induced charge corresponding to the dominant frequency of the signal increases significantly compared to the plastic stage, with the amplitude range extending to 0.5–3.16 pC.

Thus, in this uniaxial compression test, the frequency band of the available induced charge signal is 0 ~ 135 Hz, but the dominant frequency is only within 0 ~ 11 Hz.

## Quantitative relationship between damaged coal and induced charge signal

Combining the time–frequency domain features of induced charge signals, the wavelet de-noised signals are used to establish the quantitative relationship between charge accumulation and the degree of coal damage according to the statistics of coal damage.

The damage factor *D* is defined as the proportion of the failure micro-elements to the total micro-elements, which can be expressed by Eq. (10)^74^.

10 $$D = \frac{{A_{d} }}{A}$$

where *A*_*d*_ refers to the damaged area of material and *A* represents the initial area of material.

If the charge accumulation of the entire section *A* of the coal mass is *Q*_*m*_, the incidence rate of charge on per unit area *i*_*q*_ can be expressed by Eq. (11).

11 $$i_{q} = \frac{{Q_{m} }}{A}$$

When the damaged area reaches *A*_*d*_, the charge accumulation *Q*_*d*_ can be calculated by Eq. (12).

12 $$Q_{d} = i_{q} A_{d} = Q_{m} \frac{{A_{d} }}{A}$$

Substituting Eq. (12) into Eq. (10), we can get Eq. (13).

13 $$D = \frac{{Q_{d} }}{{Q_{m} }}$$

Therefore, the charge accumulation can characterize the damage factor according to Eq. (13), which reflects the degree of coal damage.

Since the press machine will stop working when reaching the pre-set damage conditions, it will result in incomplete destruction of the coal specimens, which makes it impossible for the damage factor to reach 1. As a result, the damage factor can be amended as Eq. (14)^75^.

14 $$\frac{D}{{D_{u} }} = \frac{{Q_{d} }}{{Q^{\prime}_{m} }}$$

where *D*_*u*_ is the critical value of damage and $Q^{\prime}_{m}$ is the charge accumulation when damage factor reaches the corresponding value.

The critical value of damage in Eq. (14) can be normalized with stress linearization^75^, which is obtained by Eq. (15).

15 $$D_{u} = 1 - \frac{{\sigma_{r} }}{{\sigma_{c} }}$$

where *σ*_*c*_ and *σ*_*r*_ are the peak strength and residual strength of coal specimen, respectively.

The modified damage factor *D*_*c*_ can be expressed as Eq. (16) by substituting Eq. (15) into Eq. (14).

16 $$D_{c} = \left( {1 - \frac{{\sigma_{r} }}{{\sigma_{c} }}} \right)\frac{{Q_{d} }}{{Q^{\prime}_{m} }}$$

**Figure 14:**
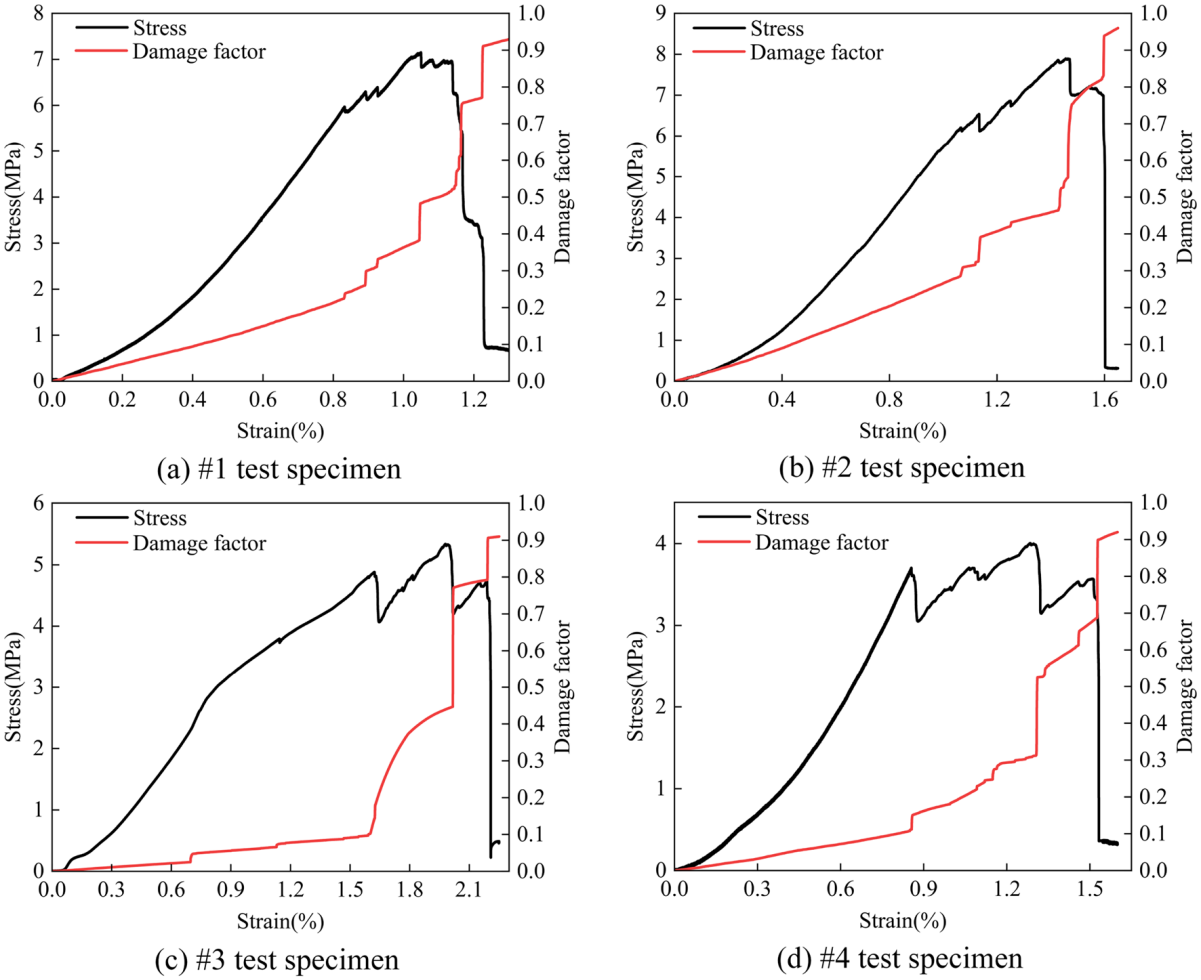
Stress-damage factor-strain curves of coal specimens under uniaxial compression.

According to Eq. (16), the curves of stress–strain and charge accumulation-strain of coal specimens under uniaxial compression conditions are as shown in Fig. 14. At the beginning of stress loading, the charge accumulation curve shows a low-amplitude linear increasing trend, and the damage degree of coal mass develops at a constant speed. During the plastic stage, the charge accumulation curve jumps in a stepwise manner, especially when the stress of the coal specimen is near the peak value. The rapid charge increase indicates that the damage degree of coal mass accelerates. Within the failure stage, the level of charge accumulation jumps the most, showing the highest degree of coal mass damage. As the coal specimen gradually approaches the residual strength point, the increased rate of charge accumulation slows down until it reaches the top, indicating that the main damage has happened.

Thus, the variation process of charge accumulation can well describe the damage and failure process of coal mass. Capturing the critical time–frequency features of induced charge signals accurately will help to evaluate the degree of the roadway surrounding rock damage.

## Discussion

The induced charge signal monitoring technique is an emerging geophysical method for monitoring the stability of roadway surrounding rock, however, it is easy to be disturbed by external interference during underground monitoring. The time–frequency features of the available charge signals are unclear in the crack emergence-expansion process of the coal mass in roadways. Thus, the effects of evaluating the roadway stability are not ideal when facing massive charge signal data. To accurately identify the degree of roadway damage, the study on time–frequency features of induced charge signals during the coal damage process is a vital problem to the success of underground applications.

### Time-domain features of induced charge signals in coal damage and failure process

The findings of the relationship between the stress and induced charge time series show the features that the damage degree of coal increases continuously with the increase of stress. In the meanwhile, the amplitude, event number, and fluctuation level of the signal increase, and strengthen the continuity, which indicates that the coal damage and failure are directly responsible for influencing the generation of induced charges. Prior studies have noted that the amplitude and density of the high-valued induced charge signals increase constantly with the increasing loads^32,35^, which proves the consistency between the intensity of induced charge signal and the degree of coal damage.

However, other studies have not yet conducted in-depth research on the time domain of induced charge signals from the perspective of coal damage. We found that different damage levels corresponding to different loading stages divided by time, different signal time-domain features are also obtained. And the internal micro-cracks development is weak during the compaction and elastic stages, with minor cumulative damage and less generated charge, resulting in unclear to identify the charge induction phenomenon. Within the plastic stage, the fluctuation, amplitude, and event numbers of available induced charge signals increase significantly, meanwhile the high-valued signals begin to appear, with the gradual transition from discrete to continuous. The most abundant signals that show continuity occur in the failure stage, with the most event numbers, the largest amplitude, and the highest degree of fluctuating. Furthermore, a few high-amplitude signals cannot recognize the degree of coal rock damage sufficiently, while a series or a large number of available charge signals occurring consecutively can better indicate that the coal medium is in a high damage and failure state.

### Frequency-domain features of induced charge signals in coal damage and failure process

In previous studies, the Fourier transform method is often used to study the frequency-domain features of the induced charge signal^35,76,77^. Fourier transform needs to determine the frequency range of the signal in advance, then select a band-pass or low-pass filter to reduce the noise of the original signal. However, the wavelet thresholding denoising method is applied in this paper, which can de-noise the original signal and is not required to grasp the accurate frequency range of the noise. The denoising accuracy of the signal is mainly dependent on the selection of the mother wavelet function^78,79^. By analyzing the signal data, the bior5.5 wavelet is chosen for signal denoising. The SNR calculation method is utilized to select the best thresholding method that is suitable for this test^80^. Moreover, using the continuous GMW transform method to analyze the frequency domain of de-noised charge signals can not only realize the corresponding analysis of the time domain and frequency domain at each loading stage but also show the time–frequency detail characteristics of the induced charge signals. It has certain advantages compared with the Fourier transform.

Through the innovative visualization of available charge signals, we find that as the degree of coal damage and failure increases, the dominant frequency center of the signal shifts towards a lower frequency direction, but the upper-frequency limit of the signal which excludes the dominant frequency is increasing continuously. In this test, the signal frequency has reached 135 Hz, and the signal duration of the dominant low-frequency is longer but the signal duration of the high-frequency is very short, which is not found in previous studies. Our findings also show that the dominant frequency of induced charge signals is extremely low, only centered at 0 ~ 11 Hz.

### Characterization of coal damage based on induced charge

Based on the key time–frequency domain features of induced charge signals, we can find that the damage of coal mass will significantly affect the signal generation. The high-value induced charge signals will be monitored due to increasing stress. During this process, the cracks begin to emerge, expand, and penetrate in the plastic stage, which will cause the crack propagation effect and triboelectric effect, accompanied by the collapse phenomenon of charged coal particles. Previous studies have shown that the charge induction laws are very closely related to the deformation damage of coal rock^67,81–83^, which proves our study.

Furthermore, the charge accumulation is used to characterize the damage degree of coal based on the damage mechanics theory. By realizing the correspondence with the curves of stress–strain and charge accumulation of the whole damage process, it verifies the correlation between coal damage and charge accumulation. According to the study results, we can find that the coal damage develops at a constant rate during the compaction and elastic stages, which shows a slow increase in the accumulated charge amount with a low range. Entering the plastic stage, each occurrence of damage and failure event is accompanied by a rapid jump in charge accumulation. Within the failure stage, the maximum jump in the charge accumulation occurs until the jump rate slows down, indicating that the main damage to the coal body has already occurred.

In recent years, some studies have proposed relating analytical relationships in the time-domain statistics of induced charge signals. Statistical indicators have clarified the correlation between induced charge and stress changes, such as post-peak charge variation rate, charge coefficient of variation^30^, and charge intensity^41^, consistent with our studies. Nevertheless, these charge indicators have not systematically established the quantitative relationship between the charge accumulation and the degree of coal damage from the perspective of damage mechanics, lacking corresponding theoretical explanations. However, in this study, the degree of coal damage is directly reflected by charge accumulation, and a complete damage characterization relationship is established to describe the damage evolution of coal mass, which can be well applied to the stability of roadway surrounding rock. In the future, additional research is needed to determine the coal damage and failure based on induced charge. With the help of the inversion of abrupt induced charge signals, the critical value of induced charge inversion is proposed, which provides a new idea for accurately identifying the precursory characteristics of coal damage and failure.

## Conclusions

This study set out to gain a better understanding of time–frequency features of induced charge signals during the damage and failure process of coal mass. Based on the free charge generation mechanism of loading coal rock and the principle of induced charge monitoring, the induced charge signal monitoring test under uniaxial compression is carried out. The critical value of available induced charge for raw signals in the experimental environment is determined, while the method for discriminating continuous or discrete induced charge signals is proposed. Combined with the wavelet analysis methods, the induced charge signals are filtered and de-noised, which contributes to investigating the time–frequency domain evolution law of the induced charge signals at each stage during the coal damage and failure. Furthermore, the quantitative relationship between coal damage and induced charge signals is established with the theory of damage mechanics. The main findings are as follows:

1. With the increase of coal damage degree, the event number, amplitude, and volatility of the induced charge signals of the coal also increase. The above three parameters of signals are all at a low level during the compaction and elastic stage, with the signal type of discrete mainly. Within the plastic stage, the event number, amplitude, and volatility of signals rise significantly, which is accompanied by the transition of signal from discrete to continuous. Eventually, the three parameters are all at the highest level in the failure stage, and the signals are mainly continuous.
2. The frequency band of the available induced charge signal after denoising during the coal damage is 0 ~ 135 Hz, but the dominant frequency is only located within 0 ~ 11 Hz, which means the available induced charge signal is basically a low-frequency signal. With the increase of damage level, the upper limit of dominant low-frequency decreases but the upper limit of high-frequency increases contrarily. The duration of the dominant low-frequency signal is accordingly longer, however, the duration of the relative high-frequency signal is shorter.
3. In the compaction and elastic stages, the available charge signals are mainly produced by the piezoelectric effect, while those in the plastic and failure stages primarily originate from the crack propagation and triboelectric by the slip and friction at the crack surface. Charge accumulation can directly reflect the degree of coal damage, and its variation process can well describe the evolution of coal damage and failure. The findings of this study imply evaluating the stability of roadway surrounding rocks.

## Data Availability

The data that support the findings of this study are available from the corresponding author upon reasonable request.
